# Present and Future of the Poor-Law Infirmary

**Published:** 1913-05-31

**Authors:** 


### PRESENT AND FUTURE OF THE POOR-LAW INFIRMARY.

### Septic Operations in Infirmaries.

Some time ago we noted the fact that a local authority had refused to sanction the establishment of a septic operating theatre in a rapidly growing infirmary, although the medical officer had strongly recommended such an innovation. The excuse was made by the Guardians that the Local Government Board did not approve of the extra expense and saw no necessity for a second operating theatre where one already existed. The incident shows clearly how difficult it sometimes is for medical men to bring to the comprehension of the lay mind those essential truths upon which all modern surgery rests, and how equally difficult it is to make local authorities understand the need for keeping their institutions up to date.

The Local Government Board does excellent service in checking unnecessary expenses and keeping down useless outlay which amounts to extravagance. But it does harm, in our opinion, in interfering between the medical man and the lay committees when the former puts in requisition for supplies which the latter may feel inclined to furnish unless the Board vetoes it. Already local authorities are very chary of furnishing the medical men with proper and up-to-date appliances, and it is easy enough to see that the objections centrally raised against the double operating theatre would appeal very forcibly to them. It is, therefore, worth while to examine these arguments briefly and ascertain how far they hold in a modern infirmary.

It is said that no special septic operating theatre is needed because such a theatre, as a special unit, is not furnished in most of our hospitals. It is a matter of fact, however, that our large hospitals possess more than one operating theatre; Guy's, with some six hundred beds, possesses eight operating rooms, irrespective of the large old theatre, which some of the senior surgeons still use. Obviously where there are so many theatres it is comparatively easy to reserve one for the use of septic cases, or to arrange cases in such a way that the septic ones are dealt with only at the end of the day's work, after which the theatre can be effectually cleaned. We admit, as a preliminary, that it is quite possible to perform a "septic operation" in a theatre, and, by means of vigorous and most careful cleansing and sterilisation, make the theatre fit for the accommodation of a "clean" case. But we should also like to state that such preparation demands time and entails considerable trouble, and that personally we would not care to be operated upon as a clean case in a theatre in which, say, half an hour before, a septic case had been treated. Moreover, it must be borne in mind that the class of septic cases in the infirmaries are in general far worse than similar cases in hospitals. There are various reasons for this; in the first instance, the infirmary gets its patient often in a very debilitated state, after he or she has been rejected by the hospitals; patients are too often very infirm and ill and weak, so that they are scarcely able adequately to resist infection by pathogenic organisms. As a result, an infection which in a clean patient, under ordinary hospital conditions, would have led, most likely, to a mild sapræmia, against which the natural powers of the individual would quickly have prevailed, turns, in the case of the old and infirm infirmary patient, to a virulent septicæmia which demands the most active and energetic surgical interference. Cases of extensive cellulitis of the face and neck and arms are comparatively common in infirmary practice; they are happily much rarer in hospital practice. Again, such cases, in hospital, are placed in a special ward; they are operated upon, invariably, in the ward itself or in an adjoining room, and are not taken into the ordinary theatre except in certain instances when amputation through clean parts is decided upon. In the infirmary such patients are in the general wards where the institution—as we know is sometimes the case—possesses no isolation room. They must be operated upon in the common theatre, with the result that the next clean case that comes into the same theatre stands a risk of getting infected with virulent pathogenic staphyllococci. Finally, consider the difference, in general, between the nursing in infirmaries which do not possess a full staff, and in hospitals where such cases may have a "special" allotted to them, and it is easy to see that the parallel between the hospital and the infirmary, in this matter of two theatres being necessary, does not hold, strictly speaking.

We maintain—and we feel sure that most infirmary workers will agree with us in this—that the need for asepsis and strict surgical cleanliness is as great in an infirmary as it is in hospital. Indeed, we will cheerfully go further and allege that while under certain conditions the strict ritual of modern surgical asepsis may be abbreviated by a hospital surgeon who works with comparatively healthy, firm-fleshed, resistant patients, the infirmary surgeon on the contrary must pay the most minute and painstaking attention to enforcing every rule of surgical cleanliness which has proved its value in the past. The latter has great difficulties to contend with. The want of a separate room—for really it is absurd to presuppose that the septic room must be as elaborately equipped and furnished as the ordinary operating theatre—in which these septic cases can be handled according to the requirements of modern surgery is one, since surgery demands in such cases deep incisions, complete evacuation of pus-containing cavities, and thorough drainage. Such a septic operating theatre is as essentially necessary in a modern infirmary as is the isolation room; where it is not provided there is reason for styling the institution which so gravely overlooks its responsibilities to patient and surgeon alike, as behind the times. The infirmary worker should therefore impress his authorities with the realities of the case. He should point out where the work is badly done, dangerously done we might say, owing to the want of isolation for the treatment of septic cases. We feel sure a few demonstrations to the authorities by the medical officers themselves will work more potently for reform than any number of recommendations or suggestions supported by no tangible evidence of danger, discomfort, and unpleasantness. Nothing is so good and effective an advocate in these cases as the nose, aided by the eye. Let a member of the committee wander into the operating theatre before and after a septic case has been operated upon, as, for instance, a bad empyema, or a case of cellulitis of the foot; there is no necessity even to let him be a witness of the operation and of the conditions found at it. If he cannot note the striking difference in atmosphere and in the general appearance of the room, he must indeed be beyond the reach of effective argument. But it is quite possible that such a demonstration, by causing his stomach to revolt, may bring home to him with the greatest possible force, the truth of the allegation made that it is dangerous and unsurgical to treat a clean case in a theatre which has just been occupied by a dirty case. And that is the crux of the whole matter. It is unreasonable to suggest that the theatre may be made sweet and clean again; it can be made so, but time and trouble are needed, and there is always a risk, even with the best cleaning. One might as well allege that no operating theatre is necessary at all, since under proper conditions the modern surgeon can operate as well in a backyard as in the best theatre. The difference lies in this, that to operate in a backyard successfully, means the expenditure of infinite pains and precautions on the part of all concerned and a supreme reliance on that vague element, surgical luck, while to operate on a patient in a modern, up-to-date theatre where proper facilities for surgical cleanliness are given means to give the patient the minimum of risk and the surgeon and nurses the maximum of convenience with the least possible effort and trouble.

